# RET Regulates Human Medullary Thyroid Cancer Cell Proliferation through CDK5 and STAT3 Activation

**DOI:** 10.3390/biom11060860

**Published:** 2021-06-09

**Authors:** Chia-Herng Yue, Muhammet Oner, Chih-Yuan Chiu, Mei-Chih Chen, Chieh-Lin Teng, Hsin-Yi Wang, Jer-Tsong Hsieh, Chih-Ho Lai, Ho Lin

**Affiliations:** 1 Department of Surgery, Tung’s Taichung Metro Harbor Hospital, Taichung 435403, Taiwan; ericchyue@gmail.com; 2 Department of Life Sciences, National Chung Hsing University, Taichung 402204, Taiwan; muhammet.oner053@gmail.com (M.O.); bradchiu19820510@gmail.com (C.-Y.C.); 3 Translational Cell Therapy Center, Department of Medical Research, China Medical University Hospital, Taichung 404332, Taiwan; midyjack@gmail.com; 4 Division of Hematology/Medical Oncology, Department of Medicine, Taichung Veterans General Hospital, Taichung 40201, Taiwan; drteng@vghtc.gov.tw; 5 Department of Life Science, Tunghai University, Taichung 40704, Taiwan; 6 School of Medicine, Chung Shan Medical University, Taichung 402, Taiwan; 7 Department of Nuclear Medicine, Taichung Veterans General Hospital, Taichung 40705, Taiwan; hywang@vghtc.gov.tw; 8 Department of Urology, University of Texas Southwestern Medical Center, Dallas, TX 75390, USA; jt.hsieh@utsouthwestern.edu; 9 Department of Microbiology and Immunology, Graduate Institute of Biomedical Sciences, College of Medicine, Chang Gung University, Taoyuan 33302, Taiwan; chlai@mail.cgu.edu.tw; 10 Ph.D. Program in Translational Medicine, National Chung Hsing University, Taichung 402204, Taiwan; 11 Rong Hsing Research Center for Translational Medicine, National Chung Hsing University, Taichung 402204, Taiwan

**Keywords:** human medullary thyroid carcinoma, CDK5/p35, RET, STAT3, ERK1/2, EGR1

## Abstract

Medullary thyroid cancer (MTC) is a neuroendocrine tumor that arises from the parafollicular C-cells, which produces the hormone calcitonin. RET is a transmembrane receptor protein-tyrosine kinase, which is highly expressed in MTC. Our previous studies reported that cyclin-dependent kinase 5 (CDK5) plays a crucial role in cancer progression, including MTC. However, the role of CDK5 in GDNF-induced RET signaling in medullary thyroid cancer proliferation remains unknown. Here, we investigated RET activation and its biochemically interaction with CDK5 in GDNF-induced medullary thyroid cancer proliferation. Our results demonstrated that GDNF stimulated RET phosphorylation and thus subsequently resulted in CDK5 activation by its phosphorylation. Activated CDK5 further caused STAT3 activation by its specific phosphorylation at Ser727. Moreover, we also found that GDNF treatment enhanced ERK1/2 and EGR1 activity, which is involved in p35 activation. Interestingly, we identified for the first time that CDK5 physically interacted with RET protein in MTC. Overall, our results provide a new mechanism for medullary thyroid cancer cell proliferation, suggesting that targeting CDK5 may be a promising therapeutic candidate for human medullary thyroid cancer in the near future.

## 1. Introduction

Medullary thyroid cancer (MTC) is a neuroendocrine tumor that arises from the parafollicular C-cells, which produces hormone calcitonin [1]. Calcitonin and carcinoembryonic antigen (CEA) are highly specific biomarkers widely utilized for the diagnosis and prognosis of MTC patients [2]. MTC accounts for 5%-10% of all thyroid cancers, and its malignancy is associated with 13% of death of patients with thyroid cancer [3]. RET is a transmembrane receptor protein-tyrosine kinase with proto-oncogene properties expressed in neural crest-derived cells such as thyroid gland [4,5]. RET mutations may result in either its abnormal activation or inhibition. RET inhibition caused by its mutation results in Hirschsprung’s disease related to hypoplasia in the central nervous system [6,7,8,9]. Likewise, MTC mainly occurs with an autosomal dominant mutation of RET, which results in abnormal RET activation. Activated RET may interact with various kinase proteins to enhance downstream signaling pathways in thyroid cancers [5,10,11]. RET mutations are associated with inducing other endocrine tumors in multiple endocrine neoplasia syndrome (MEN2A and MEN2B) [12]. MTC is mainly characterized by MEN2A and 2B mutations in the tyrosine kinase domain of the RET receptor [13]. Therefore, targeting RET proto-oncogene is an ultimate way to treat not only MTC but also non-small cell lung cancer (NSCLC). The U.S. Food and Drug Administration (FDA) has recently approved selpercatinib and pralsetinib, the first targeted therapy for cancer patients with the RET gene mutations. Selpercatinib and pralsetinib are a kinase inhibitor that blocks a type of enzyme (kinase) and prevents the proliferation of RET-altered non-small cell lung cancer (NSCLC) and certain types of thyroid cancer, including MTC [14].

In addition to RET mutations in MTC, the ligands of RET receptor protein (GDNF, neurturin, persephin, and artemin) are the glial cells line-derived neurotrophic factor family [5]. Glial cells line-derived neurotrophic factor (GDNF) is a highly conserved neurotrophic factor that promotes the survival of various types of neurons [15,16], and it signals to pass through the GDNF family receptor-α (GFRα) receptors, particularly GFRα1 to activate RET. Binding of ligands such as GDNF and other related proteins to the encoded receptor stimulates receptor dimerization and activation of downstream signaling pathways, which play essential roles in cell differentiation, growth, migration, and proliferation. RET activation by GDNF results in the subsequent activation of Ras-mitogen-activated protein kinases, PI3K, p38 and signal transducer and activator of transcription 3 (STAT3) in regulating cell proliferation and cell survival [5,17,18,19,20].

Cyclin-dependent kinase 5 (CDK5) is a proline-directed serine/threonine kinase family. Its regulatory proteins, mainly p35, activate CDK5 and its activation is highly demanded in the development of the central nervous system (CNS) [21]. Our previous studies have reported that CDK5 plays essential roles not only in the CNS, but also in aspects of cancer, including cancer cell proliferation, apoptosis, and metastasis [22,23,24,25,26,27,28,29,30,31,32]. It has been reported that medullary thyroid cancer patients with RET germline mutations show higher STAT3 activation and its nuclear localization [5]. Furthermore, our previous findings have demonstrated that CDK5 regulates STAT3 activation in the medullary thyroid cancer cell proliferation, and inhibition of STAT3 as well as CDK5 decelerates human medullary thyroid cancer cell proliferation [33]. Since CDK5 has been reported to play an essential role in various types of cancer cells, including medullary thyroid cancer cells, this study aimed to investigate the role of RET-mediated CDK5 activation and their interaction in GDNF-induced human medullary thyroid cancer proliferation.

## 2. Materials and Methods

### 2.1. Cell Culture and Treatments

Human thyroid cancer cell lines “anaplastic thyroid cancer ARO, follicular thyroid cancer WRO, papillary thyroid cancer Cg3 and medullary thyroid cancer cells TT [34,35] were kindly provided by Professor Paulus S. Wang, Department of Physiology, National Yang-Ming University (Taipei, Taiwan). All cell lines were cultured in F12 HAM medium (N3520, Sigma, St. Louis, MO, USA) supplemented with 16% fetal bovine serum (Biological Industries, Beit HaEmek, Israel), 100 Μm non-essential amino acids (NEAA), 1% sodium bicarbonate, 1% l-glutamine and 1% penicillin and streptomycin. Cells were maintained at 37 °C with 5% CO_2_ in a humidified atmosphere. TT cells were treated with GDNF (200 ng/mL) in a serum-free medium. The serum-free medium is not supplemented with FBS while supplemented with the same concentration of components mentioned above.

### 2.2. Cell Viability and Proliferation Assay

Cell viability and proliferation assay was performed as described previously [36]. Briefly, cells were plated on 24 well plates with a density of 5 × 10^4^. Treated cells with or without GDNF were counted by using hemocytometer glass each day for 6 days. The growth curve and proliferation ability of TT cells were calculated and quantified.

### 2.3. Western Blotting

Immunoblotting was performed as described previously [23,37,38]. Briefly, treated cells were collected and washed with PBS, and then the cells were lysed by lysis buffer for 45 min. Obtained cell lysates were quantified by Bradford reagent (Sigma-Aldrich, St. Louis, MO, USA) and separated by SDS-PAGE (25–40 μg/lane). After being transferred, PVDF membranes (PerkinElmer Life Sciences, Shelton, CT, USA) were blocked with 5% skim milk and then incubated with primary antibodies overnight at 4 °C. After washing with PBST, horseradish peroxidase (HRP)-conjugated secondary antibodies (Jackson Immuno Research Laboratory, West Grove, PA, USA) were incubated at room temperature. The Enhanced Chemiluminescence (PerkinElmer Life Sciences) reaction was performed, and the membranes were exposed to X-ray films (Fujifilm, Tokyo, Japan). Antibodies directed against the following proteins were used in this study: The target protein was detected by primary antibodies, including anti-Cdk5 (H-291) sc-750, anti-p35 (C-19) sc-820, anti-Ret (C-19) sc-167, anti-Egr-1(588) sc-110 from Santa Cruz Biotechnology (Dallas, TX, USA). anti-Akt (#9272), anti-phospho-Akt (Ser473) (#9271) from Cell Signaling (Danvers, MA, USA), anti-ERK1 (#610030), anti-ERK1/2 (pT202/Py204) (#612358), anti-STAT3 (#610189), phospho-STAT3 (pS727) (#612542) from BD Biosciences (San Jose, CA, USA).

### 2.4. Immunoprecipitation

Mixed Protein G Mag Sepharose Xtra beads (GE Health, North Richland Hills, TX, USA) with the specific primary antibody in a ratio of 10 μL:1 μg to make beads/antibody precipitating complex. Cellular proteins were extracted through the Cell protein extraction procedure mentioned above and incubated with beads/antibody precipitating complex at 4 °C for about 12~16 h. Using magnetic force to collect beads and analyze interacting protein of precipitated protein by western blot [30].

### 2.5. Immunocytochemistry

Cells were seeded on coverslips with a density of 2 × 10^4^. After the treatment period finished, cells were fixed with fixation buffer 4%paraformaldehydee (Sigma-Aldrich, St. Louis, MO, USA) for 15 min at room temperature. Followed by washing three times with PBS and blocking with 5% BSA in PBS, cells were incubated and co-stained with specific primary antibodies overnight. After washing three times with PBS, secondary antibodies conjugated with Alexa 488 and Alexa 546 were incubated at room temperature for 1 h. Cell nuclei were stained by DAPI fluorescent dye (Sigma-Aldrich). After further washing with PBS, slides were mounted for observation by confocal microscopy (FV1000, Olympus, Tokyo, Japan) [38].

### 2.6. siRNA Transfection

siRNA transfection was performed as previously described [27,39]. siRNA-cdk5 and nonspecific control of siRNA were purchased from Upstate (Cdk5 siRNA assay kit, 60-097, Lake Placid, NY, USA). According to the manufacturer’s instructions, the expression plasmid or siRNA were premixed within Lipofectamine 2000 or Lipofectamine RNAiMAX transfection reagent (Invitrogen, Carlsbad, CA, USA). Then, the liposome/nucleic acid complex was transfected into cells with a culture medium. Commercial products of siRNACdk5 (siCdk5) and nonspecific control siRNA (siCon) were purchased from Dharmacon (SMARTpool, Lafayette, CO, USA).

### 2.7. Statistical Analysis

Data were presented as the mean ± S.E.M. (Standard error of the mean) and paired Student’s *t*-test was used to calculate the *p*-value. Statistical significance was marked as * *p* < 0.05, ** *p* < 0.01, and *** *p* < 0.0001. No significance was marked as n.s.

## 3. Results

### 3.1. Downregulation of CDK5 Decreases GDNF-Induced Human Medullary Thyroid Cancer Cell Viability

**Figure 1:**
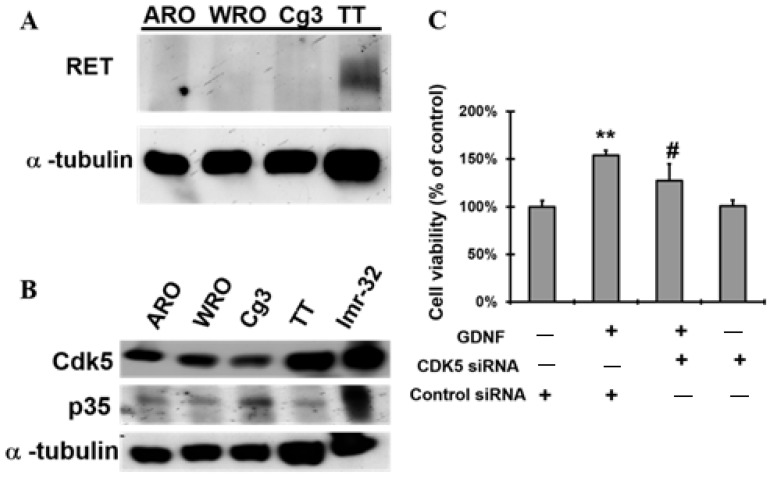
Downregulation of CDK5 decreases GDNF-induced medullary thyroid cancer cell viability in TT cells. (**A**) Protein expression level of RET in anaplastic thyroid cancer ARO, follicular thyroid cancer WRO, papillary thyroid cancer Cg3 and medullary thyroid cancer cells TT. (**B**) Protein expression level of CDK5 and p35 in ARO, WRO, Cg3, TT and Imr-32. Imr-32 (human neuroblastoma cell line) serves as a positive control for CDK5 and p35 protein, since their expression are higher in Imr-32 cells. (**C**) Cell viability of TT cells in the presence of GDNF with nonspecific control siRNA, GDNF + CDK5 siRNA, and CDK5 siRNA groups (GDNF: 200 ng/mL). Control group represents as absence of GDNF, and presence of nonspecific control siRNA. Cell viability was normalized to % of control. α-Tubulin was used as an internal control. ** *p* < 0.01, vs. control. # *p* < 0.05, vs. GDNF.

We first investigated the expression level of CDK5 and RET proteins in anaplastic thyroid cancer ARO, follicular thyroid cancer WRO, papillary thyroid cancer Cg3, and medullary thyroid cancer cells TT. Our results showed that the RET protein expression level was higher in TT cells than other types of thyroid cancer cells (Figure 1A). Regarding CDK5 with its activator protein; p35, active CDK5 is only considered with the existence of p35, thus we identified the protein expression level of both CDK5 and p35 in TT cells (Figure 1B). To determine the role of GDNF in TT cells, we performed GDNF treatment in a serum-free medium. Our results showed that GDNF treatment significantly increased TT cell proliferation in serum-free medium (Figure S1).

Next, we sought to determine whether CDK5 gets involved in the regulation of GDNF-activated RET on TT cell proliferation. Thus, we evaluated the role of CDK5 in GDNF-induced TT cell proliferation. Knock-down of CDK5 by its small interfering RNA (siRNA) transfection remarkably decreased GDNF-induced TT cell viability (Figure 1C). Nonspecific control of siRNA was used as a control. Furthermore, treatment with roscovitine (RV), a pan CDK-inhibitor, also significantly decreased the GDNF-induced TT cell proliferation (Figure S2). These data showed that inhibition of CDK5 significantly decelerated GDNF-induced TT cell growth, suggesting that CDK5 plays an important role in GDNF-induced TT cell proliferation.

### 3.2. CDK5 Physically Interacts with RET Protein in Human Medullary Thyroid Cancer Cells

**Figure 2:**
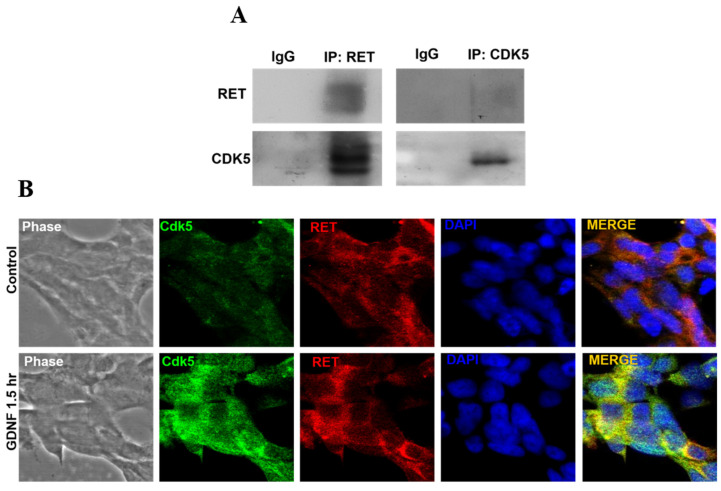

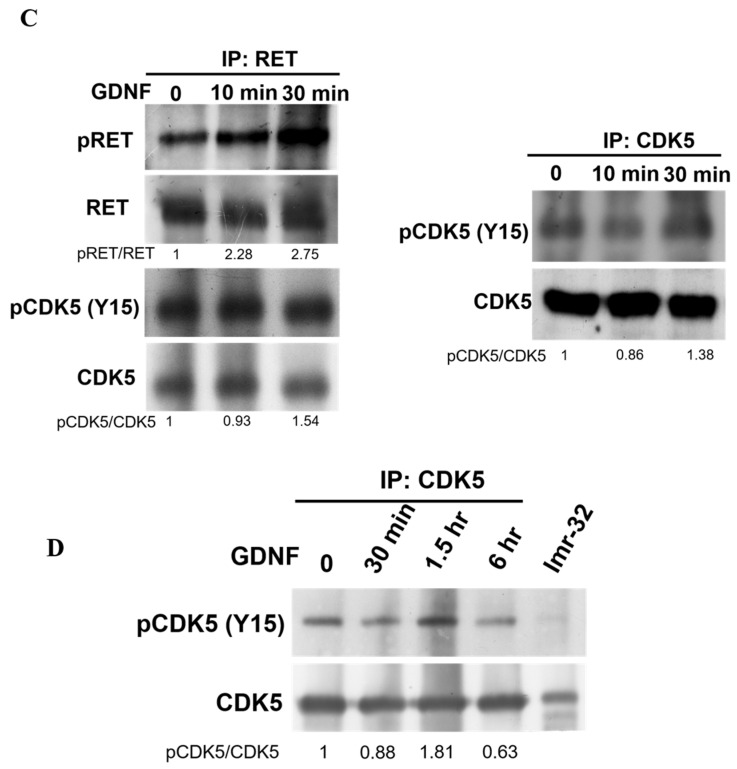
CDK5 biochemically interacts with RET protein. (**A**) Western blot data from co-immunoprecipitated RET and CDK5 protein, respectively. RET, CDK5 proteins were detected. (**B**) Immunocytochemistry data representing intracellular localization of CDK5 (green) and RET (red) proteins with the absence and presence of GDNF. Blue color indicates nucleus staining with DAPI. The yellow color in the merged image indicates the colocalization of two proteins. (**C**) Western blot data from co-immunoprecipitated RET and protein expression levels of pRET, total RET, pCDK5 and total CDK5 under GDNF treatment for 10 and 30 min, respectively. Western blot data from co-immunoprecipitated CDK5 and the protein expression level of pCDK5 and total CDK5 with GDNF treatment for 30 min, 1.5 h and 6 h, respectively. (**D**) Western blot data from immunoprecipitated CDK5 and immunoblotting CDK5 and pCDK5 (Y15) under time-dependent GDNF treatment. Imr-32 (human neuroblastoma cell line) serves as a positive control for CDK5 and p35 protein, since their expression are higher in Imr-32 cells.

It has been well established that RET, as a transmembrane protein, interacts with several tyrosine kinase proteins, and their intracellular interaction has been associated with various type of cancer cell proliferation [40,41,42]. Since CDK5 and RET have been reported to play an essential role in human medullary thyroid cancer cell proliferation, we next hypothesized that there might be some regulation between CDK5 and RET proteins. Therefore, we investigated the protein interaction between RET and CDK5 in TT cells. Our data showed that CDK5 physically interacted with RET protein in TT cells (Figure 2A). Protein interaction between RET and CDK5 was further confirmed by evaluating their co-localization in human medullary thyroid cancer cells. Our results showed that CDK5 co-localized with RET protein, and GDNF treatment induced CDK5 and RET protein interaction mainly at the cell membrane (Figure 2B). These data demonstrated that physical interaction between CDK5 and RET might be associated with GDNF-induced human medullary thyroid cancer growth. To determine the activation status of RET and CDK5 after GDNF treatment, we next evaluated the expression level of CDK5 and RET proteins with a time-dependent GDNF treatment in TT cells.

We first confirmed that GDNF treatment increased RET activation in 30 min by its phosphorylation. However, CDK5 activity was not increased by short term GDNF treatment in both immunoprecipitated RET and CDK5 group (Figure 2C). To observe the CDK5 activity induced by GDNF, we performed long-term GDNF treatment in the immunoprecipitated CDK5 group. Our results showed that CDK5 phosphorylation in 1.5 h increased by GDNF treatment (Figure 2D).

These data suggested that GDNF-activated RET protein recruited CDK5 protein and consequently induced its phosphorylation and activation. Since we found that GDNF induced physical interaction between RET and CDK5 protein, which resulted in a CDK5 activation by its phosphorylation, we wondered whether intracellular localization of CDK5 was affected by time-dependent GDNF treatment. Our results showed that GDNF treatment altered the intracellular localization of CDK5. We found that GDNF induced intracellular localization of CDK5 mainly at the cell membrane (Figure S4), suggesting that GDNF-induced RET activation recruits CDK5 by altering its intracellular localization through the cell membrane to induce the activation of CDK5.

### 3.3. Downregulation of CDK5 Inhibits GDNF-Induced STAT3 Activation in Human Medullary Thyroid Cancer Cells

**Figure 3:**
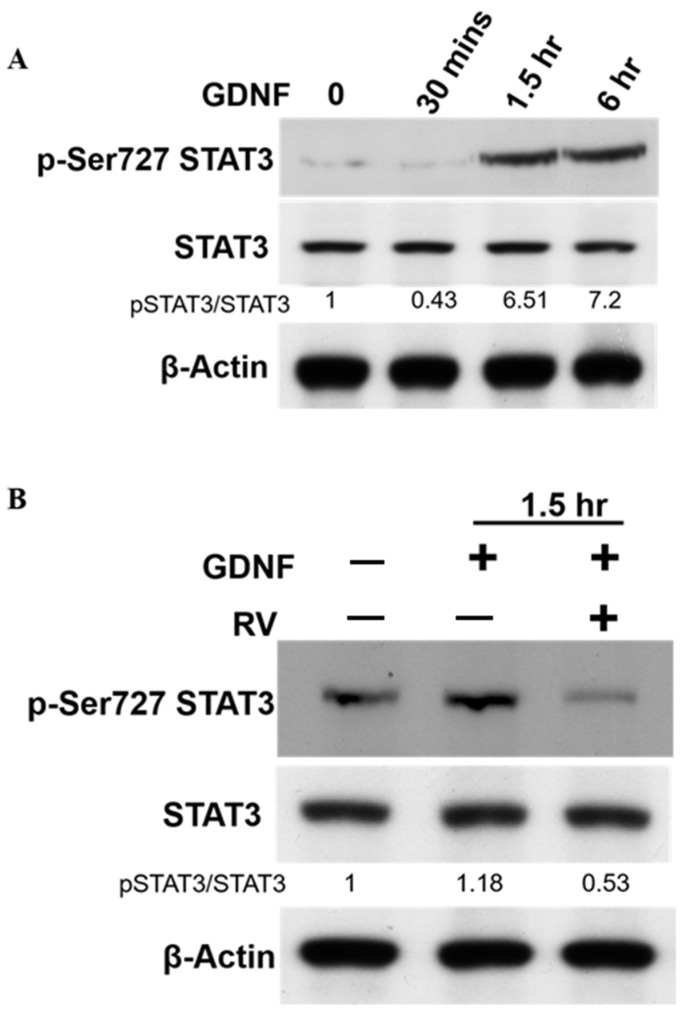
Downregulation of CDK5 inhibits STAT3 activation. (**A**) Protein expression level of pSTAT3 (ser727) and total STAT3 proteins under GDNF treatment for 30 min, 1.5 h and 6 h, respectively. (**B**) Protein expression level of pSTAT3 (ser727) and total STAT3 proteins under the treatment of GDNF, and GDNF + RV for 1.5 h. RV: Roscovitine, a pan CDK inhibitor.

Our current data demonstrated that CDK5 and its subunit p35 play an essential role in GDNF-activated RET signal transduction. Based on these findings, we next investigated the downstream of CDK5 in this signal transduction. Since we have previously reported that CDK5 phosphorylates STAT3 at Ser727 site in prostate and medullary thyroid cancer cells [22,28,33], thus, we thought that STAT3 might be a potential downstream of CDK5 in GDNF-induced RET signaling in medullary thyroid cancer cell proliferation. We evaluated the phosphorylation of STAT3 and its total protein expression under GDNF treatment. Our results showed that GDNF treatment increased Ser-727 phosphorylation of STAT3 in 1.5 h while did not affect total STAT3 (Figure 3A). STAT3 activation status showed similar activation status with CDK5 (Figure 2D), suggesting that STAT3 activation might be CDK5-dependent in GDNF-induced medullary thyroid cancer cell proliferation. To clarify this phenomenon, we next investigated the inhibition of CDK5 and observed the Ser-727 phosphorylation of STAT3. Our data showed that CDK5 inhibition by a CDK inhibitor called roscovitine (RV) decreased the GDNF-induced Ser-727 phosphorylation of STAT3 (Figure 3B). These data confirmed that GDNF-mediated STAT3 activation depends on CDK5 activity in human medullary thyroid cancer cells. Thus, STAT3 might be the helper of p35/CDK5 to pass RET signaling to the nucleus.

### 3.4. GDNF-Induced p35/CDK5 Activity Is ERK-Egr1-Dependent in Human Medullary Thyroid Cancer Cells

**Figure 4:**
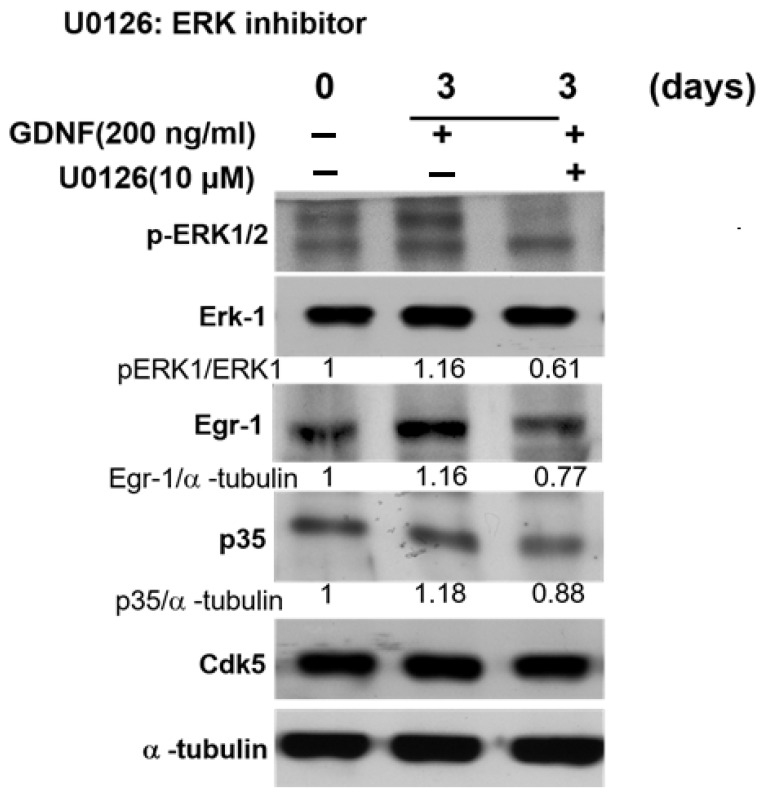
GDNF-induced p35/CDK5 activity is ERK-EGR1 signaling-dependent. The protein expression level of pERK1/2, ERK1, EGR1, p35 and CDK5 under GDNF and ERK inhibitor (U0126) for 3 days.

Our previous findings demonstrated that nerve growth factor (NGF), a neurotrophic factor, induced p35/CDK5 activity through ERK-Egr1 activation during neuronal differentiation [32]. Since our current data also showed that GDNF enhanced p35/CDK5 activity in TT cell, therefore, we investigated similar signaling pathways that might be related to CDK5 activation in medullary thyroid cancer cell proliferation. For this purpose, we performed 4 days of GDNF treatment to observe the proliferation status of TT cells and signal mechanism. Our results demonstrated that GDNF induced the phosphorylation of ERK1/2 in 3 days of treatment. Increased phosphorylation of ERK protein caused Egr1 activation, which resulted in an increased p35 expression in 3 days of treatment (Figure S3). To confirm the role of ERK-Egr1 signaling pathway in GDNF-mediated RET signaling through CDK5 activation in TT cells, we next performed ERK1/2 inhibition by its specific inhibitor U0126. Our results showed that ERK protein inhibition decreased Egr1 and p35 protein expression levels (Figure 4). These data confirmed that GDNF-mediated p35/CDK5 activation is ERK and Egr1 activation-dependent in medullary thyroid cancer cell proliferation.

## 4. Discussion

RET is a transmembrane protein consist of the intracellular tyrosine kinase domain, and therefore it is classified in the receptor tyrosine kinase (RTK) family. Classic RTK activation occurs when the specific ligands bind to its receptor [43]. As observed in RTK activation, RET activation is also required to bind with its ligands, mainly glial cell-derived neurotrophic factor family (GFLs) to its co-receptor (GFRα). GFL and GFRα complex phosphorylates RET protein by binding to its extracellular domain. RET activation results in activating the various signaling pathway, including PI3K, JAK-STAT, and PKA [42]. The mutations that occurred in RET proto-oncogene are essential in regulating tumorigenesis. These genetic mutations in RET are observed in hereditary and sporadic medullary thyroid cancer (MTC) progression [44]. The hereditary form originates with the multiple endocrine neoplasia (MEN) disorder. MEN syndrome is characterized as MEN1, MEN2A, and MEN2B. The MEN1 gene mutations are associated with tumor progression. Particularly, MEN2A mutations are strongly associated with medullary thyroid cancer, pheochromocytoma, and hyperparathyroidism. MEN2B has similar features to MEN2A. Additionally, MEN2B is correlated with different hormonal disorders [43,45,46,47]. Oncogenic RET mutations in patients with MTC is the main feature of tumor growth. RET germline mutations in non-cysteine codons have been reported as the most common mutations occurring with MEN2 [48]. Somatic RET mutations have been described in sporadic MTC. Particularly, M918T has been associated with poor prognosis and tumor metastasis in patients with somatic RET mutations [44,49]. It is well known that kinase proteins can regulate RET protein. Therefore, multiple kinase inhibitors targeting RET protein have been actively utilized to treat non-small cell lung cancer (NSCLC) and MTC [50,51]. Regarding CDK5, it is an important member of the serine/threonine kinase family [52]. Therefore, we thought that CDK5, as a kinase protein, might affect RET protein and help pass signaling to the nucleus in MTC proliferation. CDK5 is activated by its subunits and is expressed in postmitotic neurons. CDK5/p35 is an essential protein complex that regulates multiple cellular processes, including neuronal development, neuronal differentiation, and neurodegeneration.

In addition to the role of CDK5 in the CNS, CDK5 has been reported to play essential roles in cancer progression. Our previous studies indicated that CDK5 promotes prostate cancer cell growth in vitro, in vivo, and prostate cancer patients [22,23,53]. Since it has been reported that CDK5 can interact with several important proteins in cancer progression [22,23,53,54,55], we also demonstrated that CDK5 has a direct protein-protein interaction with STAT3 in prostate cancer cell growth [22,28]. Although CDK5 has been reported as an oncogenic protein in various cancer types, the opposite results have also been reported in gastric cancer [56,57]. Regarding neuroendocrine features of cancers, neuronal cells and neuroendocrine cancers originate from the neural crest. CDK5 plays essential roles for the neuronal cells; therefore, CDK5 may be a promising target in treating neuroendocrine tumors. Regarding the active role of CDK5 in cancer progression, we have previously identified the cumulative roles of CDK5 in neuroendocrine cancers, including medullary thyroid cancer [22,23,24,25,26,28,33]. Since p25 is a more stable and potent activator for CDK5 relative to p35, therefore, we previously investigated the expression level of CDK5 activator proteins, including p35 and p25. Our previous data showed that p35 and CDK5 protein expression levels were higher in human TT cells. Interestingly, lower p25 was observed in human TT cells. To identify the role of p25 in MTC proliferation, we thought that inhibition of p35 by siRNA might not be an appropriate approach because it may result in the inhibition of both p35 and p25 at the same time. Thus, we utilized the overexpression of p25 protein to evaluate MTC cell proliferation. Our previous data revealed that the overexpression of p25 did not affect MTC proliferation, although CDK5 activation was observed [33]. These data suggest that p35, but not p25, plays an important role in maintaining MTC proliferation. Furthermore, it has also been reported that overexpression of p25 deregulates CDK5 activity [58]. These findings suggest that MTC may originate from aberrant CDK5 activation as much as RET mutations. Therefore, we hypothesize CDK5 might play important roles in MTC proliferation by regulating RET protein. We first investigated the expression of CDK5 and p35 in anaplastic thyroid cancer ARO, follicular thyroid cancer WRO, papillary thyroid cancer Cg3, and medullary thyroid cancer cells TT. Our results indicate that CDK5 is highly expressed in TT cancer cells relative to other thyroid cancer cell lines. After we confirmed that CDK5 and p35 expression level was higher in TT cells; next, we focused on TT cells to evaluate CDK5 and RET interaction. To induce MTC proliferation through RET phosphorylation and activation, we performed GDNF stimulation on human TT cell line and evaluated its proliferation status through CDK5 and p35 activation. Our current results show that GDNF stimulation remarkably induces MTC proliferation.

**Figure 5:**
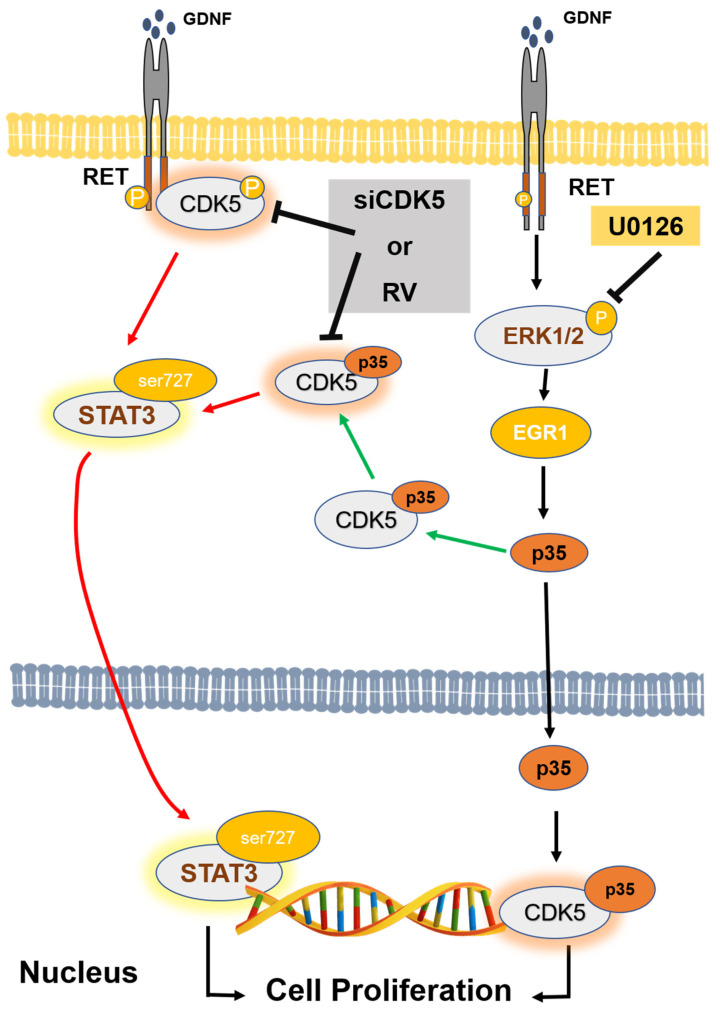
A schematic illustration of signaling mechanism in GDNF-induced human medullary thyroid cancer cell proliferation. GDNF activates RET protein. Activation of RET causes phosphorylation of ERK1/2 and subsequent activation of EGR1. Active EGR1 causes nuclear accumulation of p35 that can binding and activates CDK5. Active CDK5 physically interacts with the intracellular domain of RET protein to pass through the GDNF-activated signaling cascade. Finally, CDK5 activates STAT3 by its specific phosphorylation on Ser727. This further trigger medullary thyroid cancer cell proliferation.

Next, we asked whether CDK5 plays a regulatory role in GDNF-induced MTC proliferation. To determine the function of CDK5, we performed CDK5 inhibition by using siRNA or its inhibitor, roscovitine. Our data demonstrate that CDK5 inhibition significantly prevents GDNF-induced MTC proliferation and cell viability. This data confirmed that CDK5 plays an important role in GDNF-induced MTC proliferation. Next, we asked whether CDK5 gets involved in RET signaling after GDNF stimulation. For this purpose, we performed immunoprecipitation to evaluate protein-protein interaction in TT cells. Interestingly, we identified for the first time that CDK5 physically interacts with RET protein in GDNF-induced medullary thyroid cancer cell proliferation. Our results reveal that CDK5 can bind intracellular kinase domain of RET protein in TT cells. Moreover, our results demonstrate that CDK5 protein co-localizes with RET protein mainly at the cell membrane. These data suggest that RET activation may recruit CDK5, and it may activate CDK5 to stimulate CDK5-mediated downstream signaling cascade. To observe the activation status of RET and CDK5 protein, we performed time-dependent GDNF stimulation. Our results show that short term GDNF stimulation can only increase phosphorylation of RET protein. Besides, in this short-term treatment, GDNF stimulation induces the protein-protein interaction between CDK5 and RET, although GDNF could not induce the phosphorylation of CDK5. Therefore, to observe the alteration of CDK5 phosphorylation, we performed GDNF stimulation for long time. GDNF stimulation increases CDK5 phosphorylation on Y15 after 1.5 h of treatment. Next, we asked whether time-dependent GDNF stimulation alters the colocalization between CDK5 and RET in TT cells. Our data showed that GDNF treatment enhanced the colocalization between CDK5 and RET protein mainly at the cell membrane. Overall, our data reveal that CDK5 activation occurs after RET activation by GDNF stimulation, suggesting that CDK5 may be a potential downstream of RET protein in GDNF-induced human medullary thyroid cancer cell proliferation. After understanding the physical interaction between CDK5 and RET, the next question is how this interaction passes their signal into the nucleus in GDNF-induced MTC proliferation. We first investigated that GDNF treatment enhanced nuclear localization of p35 protein in time dependent-manner, suggesting that CDK5 and p35 may localize in the nucleus and result in an enhanced MTC proliferation. Direct nuclear localization of p35 may be one explanation of how RET signaling goes to the nucleus in MTC proliferation. Besides, our previous study demonstrated that CDK5 phosphorylates STAT3 in prostate and medullary thyroid cancer cells [22,28,33]. Since several studies have reported that RET activates STAT3 in different types of cancer [59,60,61,62], CDK5-mediated STAT3 activation with GDNF treatment remains unknown in this study. Therefore, we next evaluated STAT3 activation status in GDNF-induced MTC cell proliferation. Our current data demonstrated that GDNF stimulation increased CDK5 phosphorylation, resulting in STAT3 activation, thus further triggering MTC cell proliferation. Besides, GDNF-induced STAT3 phosphorylation on Ser727 was inhibited by a specific CDK inhibitor, RV, suggesting that CDK5 might be upstream of STAT3 protein to pass the signal to the nucleus in GDNF-induced MTC cell proliferation. Activated STAT3 by CDK5 may be another possible explanation of how RET signaling passes to the nucleus in MTC proliferation. Furthermore, our previous study reported that neurotrophic growth factors such as nerve growth factor induce neuronal differentiation in rat pheochromocytoma cells through ERK and EGR1 activation, associated with p35/CDK5 activation [32]. We asked whether a similar signaling pathway may get involved in our current study based on these findings. Therefore, we performed a long-time GDNF stimulation to observe the proliferation status of TT cells. Interestingly, we found that long term GDNF treatment remarkably enhances phosphorylation of ERK1/2 protein, and consequently increases expression level of EGR1 and p35. Thus, ERK and EGR1 signaling pathway may be responsible for the CDK5 activation in GDNF-induced MTC proliferation. To clarify this question, we performed ERK inhibition by its specific inhibitor. ERK inhibition causes a decreased phosphorylation of ERK1/2, and decreased expression level of EGR1. Moreover, ERK inhibition also reduced expression level of p35 protein. These data confirmed our hypothesis that ERK and EGR1 signaling play important roles in regulating CDK5 expression in neuroendocrine thyroid cancer proliferation (Figure 5). ERK and EGR1 activation may be an upstream regulator of CDK5 protein, therefore, it may be another explanation of how RET signaling passes to the nucleus through CDK5 activation.

## 5. Conclusions

In conclusion, our study reveals that CDK5 plays an essential role in regulating GDNF-induced human medullary thyroid cancer cell proliferation. CDK5 inhibition remarkably reduces cell viability and cell proliferation in human medullary thyroid cancer. In this study, we identified for the first time that CDK5 physically interacts with RET transmembrane proto-oncogene protein. This interaction results in the subsequent STAT3 activation to promote human medullary cancer cell growth (Figure 5). Considering the malignancy of medullary thyroid cancer, patients with advanced and metastasized medullary thyroid cancer are difficult to treat since tumor cells do not respond to chemotherapeutic treatment and external radiation [63,64,65]. Therefore, targeting of CDK5 might be a therapeutic approach for the treatment of human medullary thyroid cancer in the near future.

## Supplementary Materials

The following are available online at https://www.mdpi.com/article/10.3390/biom11060860/s1, Figure S1: GDNF accelerates TT cell proliferation. Figure S2: CDK5 inhibition decelerates TT cell proliferation. Figure S3: GDNF-induced p35/CDK5 activity is ERK-EGR1 dependent. Figure S4: GDNF alters intracellular localization of CDK5 mainly at cell membrane.
