# Supplementary material for: RET Regulates Human Medullary Thyroid Cancer Cell Proliferation through CDK5 and STAT3 Activation
Source: Biomolecules. 2021 Jun 9;11(6):860. doi: 10.3390/biom11060860 (PMC8229599; doi:10.3390/biom11060860)
Supplement: Supplementary file 1 [file biomolecules-11-00860-s001.zip › biomolecules-1219194-supplementary.pdf]

Figure S1:

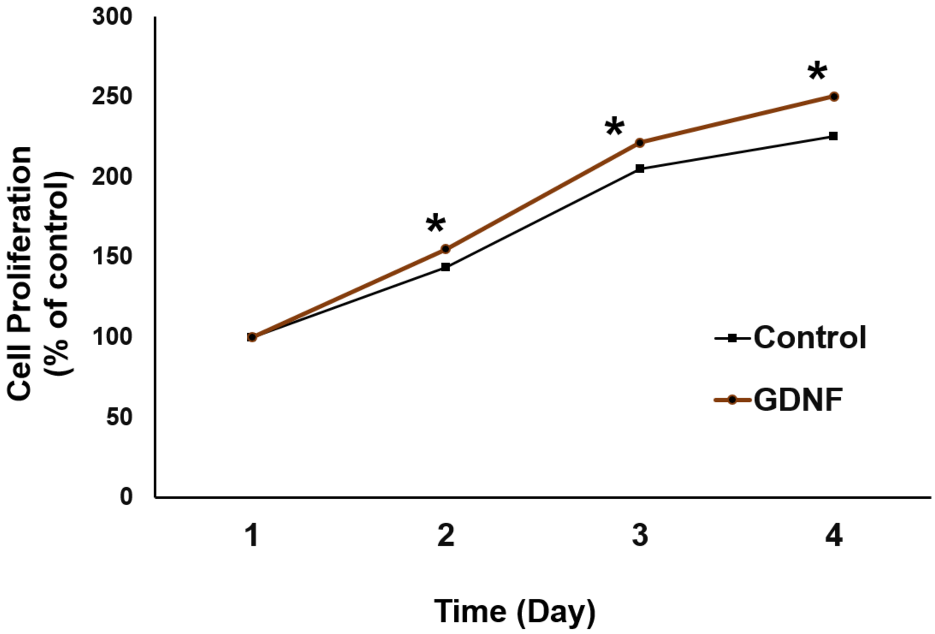

FigureS1: GDNF induces TT cell proliferation with a time dependent manner. Treatment with 200ng/ml GDNF significantly induces TT cell proliferation in serum-free condition.

Figure S2:

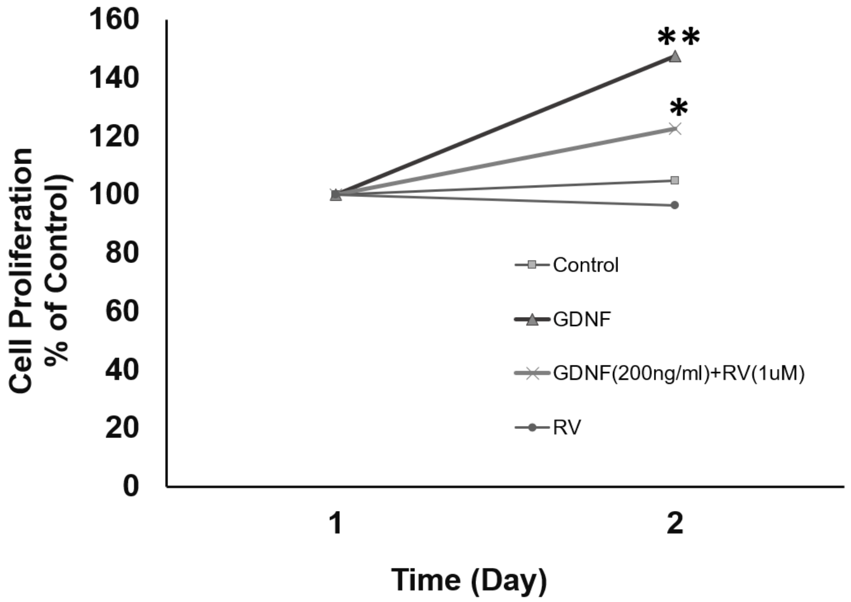

FigureS2: Inhibition of CDK5 reduced GDNF-induced TT cell proliferation. CDK5 inhibition by RV significantly inhibited GDNF-induced TT cell proliferation.

Figure S3:

A

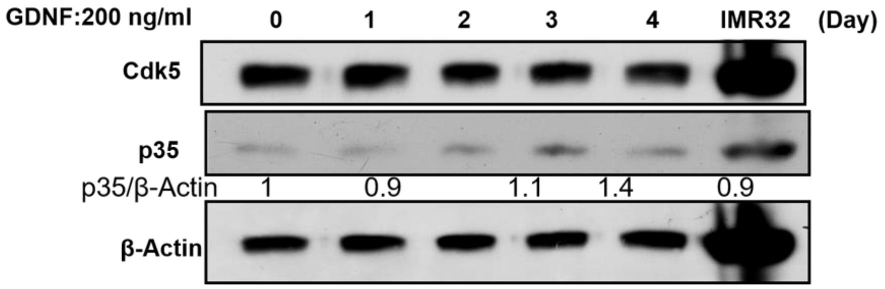

B

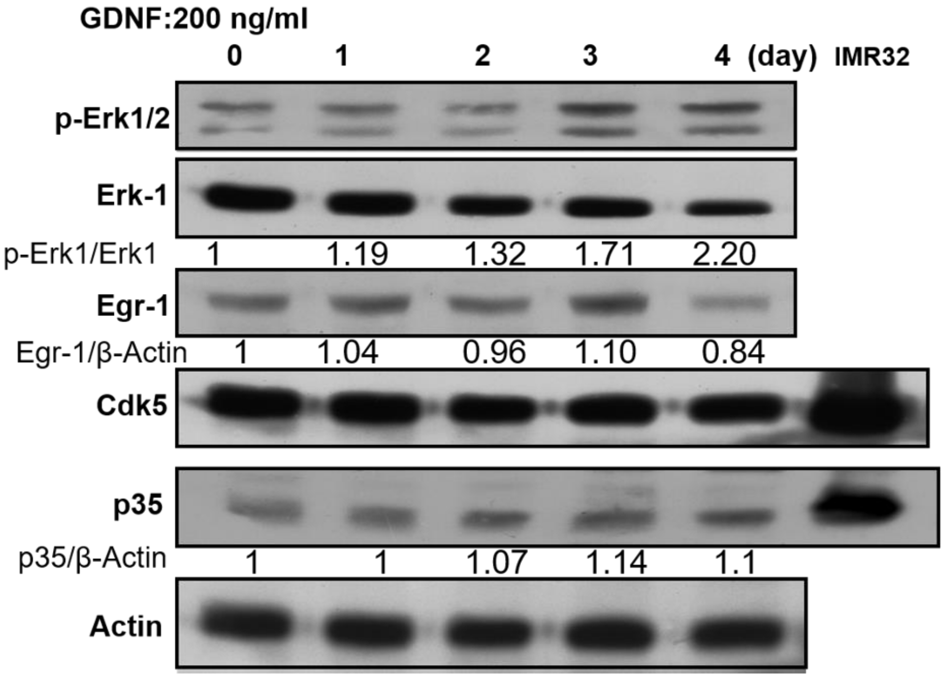

FigureS3: GDNF-induced RET activation regulates ERK1-EGR1 signaling to promote p35/CDK5 complex. We performed 4 days of GDNF treatment to observe the proliferation status of TT cells and signal mechanism. Our results demonstrated that GDNF induced the phosphorylation of ERK1/2 in 3 days of treatment. **(A)** western blot image of CDK5 and p35 under GDNF treatment for 4 days. **(B)** Western blot image of pERK1/ERK1, EGR1, p35 and CDK5.

Figure S4:

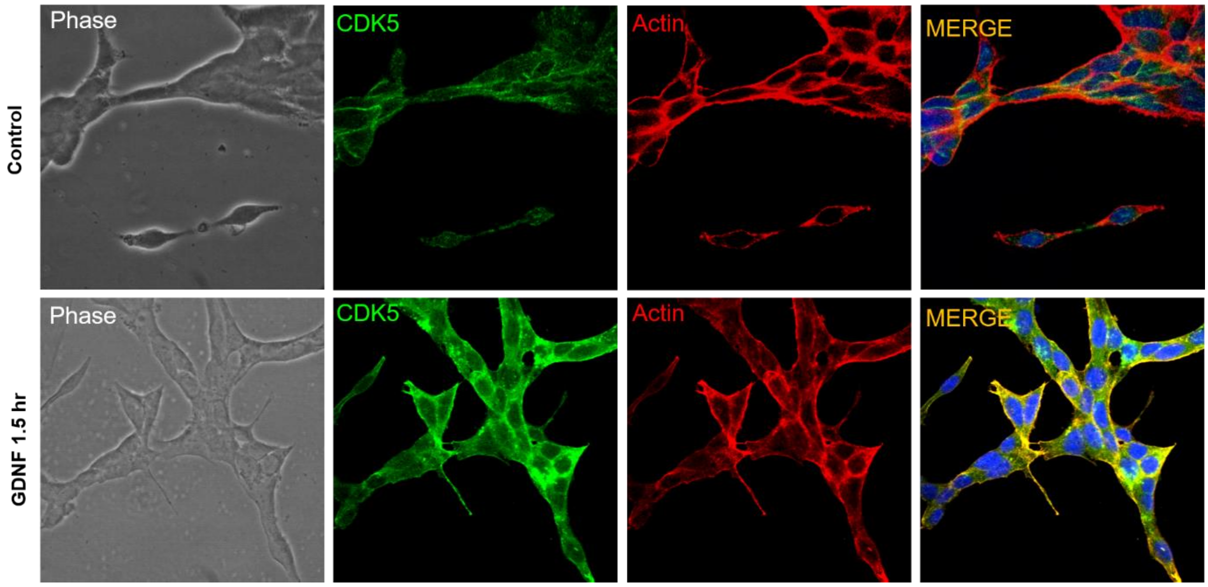

Figure S4: Immunocytochemistry showing intracellular localization of CDK5 with or without GDNF treatment. CDK5 (green) mainly localizes at cell membrane after 1.5 hours of GDNF treatment. Actin (red) was used to label cell cytoskeletons to identify cell membrane with the analyzing multiple sections of cell layers by confocal microscope. Yellow color indicates co-localization between CDK5 and Actin at cell membrane.
